# A Fish Eye Out of Water: Ten Visual Opsins in the Four-Eyed Fish, *Anableps anableps*

**DOI:** 10.1371/journal.pone.0005970

**Published:** 2009-06-24

**Authors:** Gregory L. Owens, Diana J. Windsor, Justin Mui, John S. Taylor

**Affiliations:** Department of Biology, University of Victoria, Victoria, British Columbia, Canada; University of Edinburgh, United Kingdom

## Abstract

The “four-eyed” fish *Anableps anableps* has numerous morphological adaptations that enable above and below-water vision. Here, as the first step in our efforts to identify molecular adaptations for aerial and aquatic vision in this species, we describe the *A. anableps* visual opsin repertoire. We used PCR, cloning, and sequencing to survey cDNA using unique primers designed to amplify eight sequences from five visual opsin gene subfamilies, SWS1, SWS2, RH1, RH2, and LWS. We also used Southern blotting to count opsin loci in genomic DNA digested with *EcoR1* and *BamH1*. Phylogenetic analyses confirmed the identity of all opsin sequences and allowed us to map gene duplication and divergence events onto a tree of teleost fish. Each of the gene-specific primer sets produced an amplicon from cDNA, indicating that *A. anableps* possessed and expressed at least eight opsin genes. A second PCR-based survey of genomic and cDNA uncovered two additional LWS genes. Thus, *A. anableps* has at least ten visual opsins and all but one were expressed in the eyes of the single adult surveyed. Among these ten visual opsins, two have key site haplotypes not found in other fish. Of particular interest is the *A. anableps*-specific opsin in the LWS subfamily, S180γ, with a SHYAA five key site haplotype. Although *A. anableps* has a visual opsin gene repertoire similar to that found in other fishes in the suborder Cyprinodontoidei, the LWS opsin subfamily has two loci not found in close relatives, including one with a key site haplotype not found in any other fish species. *A. anableps* opsin sequence data will be used to design *in situ* probes allowing us to test the hypothesis that opsin gene expression differs in the distinct ventral and dorsal retinas found in this species.

## Introduction

*Anableps anableps* is an active surface feeder found in the murky intertidal regions, oceanic shore waters, and freshwater streams of Central America and northern South America [1]. It can jump out of water to catch flying insects, but also feeds on floating material and diatoms in riverbank mud [2]. *A. anableps* eyes have morphological adaptations that allow for simultaneous vision above and below water. For example, its cornea is separated into two parts by a pigment stripe that prevents glare [3], with the above-water portion flatter than its ventral counterpart [4]. This difference appears to compensate for the fact that light entering the cornea from the aerial environment is refracted much more than light entering from the aquatic environment [5]. While most fish have spherical lenses, in *A. anableps* the lens is oval-shaped. This allows light from the aerial field to pass through a relatively flat portion of the lens, similar to the lens of a land animal, and light from the aquatic environment to pass through a portion of the lens with a curvature more typical for an aquatic animal [4]. Finally, the retina is divided into dorsal and ventral portions, which receive light from the aquatic and aerial environment respectively.

There are two other species in the genus *Anableps*, *A. microlepis* (the finescale four-eyed fish), which is found on the Atlantic coasts of Central and South America, and *A. dowei* from the Pacific coast of Central America. All three *Anableps* species possess the unusual eye morphology. The other taxa in the family Anablepidae, genus *Jenynsia* (sister taxon to *Anableps* with 12 species) and genus *Oxyzygonectes* (with one species *O. dovii*), have typical teleost eyes with a single cornea and pupil, a spherical lens, and a cup-shaped retina [6].

Beyond morphology, vision can also be examined at a molecular level. Light receptors expressed in rod and cone cells of the retina are called opsins. Each opsin protein is associated with a chromophore and when exposed to light this complex changes conformation leading to rod or cone cell hyperpolarization [7]. The detection of light requires input from just one type of opsin-chromophore receptor. However, no single opsin receptor is sensitive to all wavelengths of visible light. Furthermore, wavelength discrimination (colour vision) involves the interpretation of signals from different subpopulations of cone cells expressing opsins with different spectral sensitivities [8].

Gene duplication and divergence events are the evolutionary source of opsins with different spectral sensitivities. In vertebrates there are SWS1, SWS2, RH2, and LWS cone opsins. SWS opsins are Short Wavelength Sensitive opsins that are most sensitive to UV and blue light. RH2 opsins (Rhodopsin-like) are most sensitive to wavelengths in the middle of the visible light spectrum (i.e. green light) and LWS (Long Wavelength Sensitive) opsins are most sensitive to orange and red light. Rod cells, which function primarily in dim light, express RH1 genes (Rhodopsin) that encode a green light absorbing pigment [9]. Opsin subfamilies have been expanded or lost in different vertebrate lineages. For example, while dogs have one LWS opsin, humans have two, and guppies (*Poecilia reticulata*) have four [10], [11]. Placental mammals have lost both the RH2 and SWS2 opsin subfamilies and the coelacanth (*Latimeria chalumnae*) has lost all but the RH1 and RH2 opsins [10], [12].

Individual opsins vary in their spectral sensitivity among and within subfamilies. This variation is a result of changes at key amino acid sites, which are sites that have a disproportional effect on spectral sensitivity and are often found at locations where the opsin contacts the chromophore [13]. Previous work has quantified the contribution of each site to the overall wavelength of maximal sensitivity (λ_max_) and it is therefore possible to identify opsins within subfamilies with different spectral sensitivities by comparing their amino acid sequences. In fish there are two types of opsin-associated chromophore, A1 and A2, and depending on which is used the spectral sensitivity can differ by up to 50 nm [14]. Some species tune their vision by switching from one chromophore to the other in response to environmental or developmental changes [15], [16]. While chromophore use is not considered here, previous study has shown homogenous use of A1 in the *A. anableps* retina [17].

Microspectrophotometry (MSP) is a technique that estimates wavelength sensitivity (λ_max_) at the cellular level. An MSP study detected only three different classes of cones cells in *A. anableps* [17]. However, phylogenetic data from close relatives, guppy and bluefin killifish (*Lucania goodei*), suggests that it has many more. MSP data might not reflect the four-eyed fish's true repertoire if only a subset of loci are expressed in adults or if multiple opsins are expressed in the same photoreceptor, as has been shown in mice, eels, and salamanders among others [18]–[20].

In addition to gene number and sequence, opsin expression also varies among species, populations and even within individuals at different periods of development. For example, in cichlids LWS opsin expression varies with water turbidity and it appears that population-level variation in wavelength sensitivity has played a role in variation in male colouration [21]. At the individual level, European eels (*Anguilla anguilla*) have two RH1 opsins, each tuned to slightly different wavelengths. They express a green-shifted locus as juveniles in fresh water and a paralogous blue-shifted locus when they return to the ocean [22]. The lamprey (*Geotria australis*) also adjusts its spectral sensitivity by switching from the expression of one opsin paralog to another as it moves between marine and freshwater environments and Zebrafish (*Danio rerio*) have two LWS opsins that are expressed at different times of development and in different regions of the retina [23], [24]. Given these observations, we hypothesized that the morphological adaptations leading to simultaneous aerial and aquatic vision in *A. anableps* would be accompanied by changes in opsin gene number and/or sequence and by changes in opsin expression patterns.

Here we report the results of a PCR-based survey of *A. anableps* opsins using primers complementary to regions of each locus that are conserved in closely related species. We also used Southern blotting probes to identify the number of opsin loci in the *A. anableps* genome. These techniques revealed that *A. anableps* has ten visual opsins, including representatives from each opsin subfamily.

## Results

### Visual opsin sequences

Transcripts of eight opsin genes (SWS1, SWS2A, SWS2B, RH2-1, RH2-2, RH1, LWS S180α, and LWS S180r) were amplified and sequenced using primers listed in Table 1 from cDNA derived from a single *A. anableps* eye. Southern blotting experiments utilized LWS, SWS1, SWS2, RH2 and RH1 opsin gene probes and two samples of *A. anableps* genomic DNA, one digested with *EcoR1* and the other digested with *BamH1*. These experiments indicated there might be two SWS1 and RH1 loci and three LWS loci (Figure S1, Table 2). We used PCR to survey *A. anableps* genomic DNA to test the hypothesis that there were additional loci in these three subfamilies not detected in cDNA. Five clones with inserts derived from RH1-specific primers and five clones with inserts derived from SWS1-specific primers were sequenced and all had the same sequence as the original cDNA amplicon.

**Table 1:** Primers used for cDNA and genomic PCR and Southern blot probe synthesis.

Opsin category	Primer Name	Sequence
SWS1	SWS1Fw1	5′- AACTACATCYTGGTMAACATCTCC-3′
	SWS1Fw2	5′- TGGGCSTTCYACCTGCAGGC -3′
	SWS1Rev1	5′- GAGTAGGAGAARATGATGATGG-3′
	SWS1Rev2	5′-GAACTGTTTGTTCATGAAGGCG-3′
SWS2	SWS2Fw1	5′-GYACWATTCAATACAAGAARC-3′
	SWS2Fw3	5′-AGCCTTTGGTCTCTGGCTGTG-3′
	SWS2Rev1	5′-AAAGCARAAGCAGAAGAGGAAC-3′
	SWS2Rev4	5′-CCCGTTGTGTACCAGTCTGG-3′
	SWS2AFw1	5′-GTCCACCCGAGTCATAGAGC-3′
	SWS2ARev2	5′-GCCCACGGTTGTTGACAAC-3′
	SWS2-2Fw2	5′-TCTACACCATGGCTGGATTCAC-3′
	SWS2-2Rev1	5′-GATGGTGGTGAATGGAACAGC-3′
RH2	RH2Fw1	5′- AACTTCTAYATCCCGWTGTCC-3′
	RH2Fw2	5′-TGHTCTTCCTGATCTKCACTGG-3′
	RH2Rev2	5′-GTCTCRTCCTCCACCATGC-3′
	RH2Rev4	5′- TGCGGCATGAGTTCCAGTG-3′
	RH2-2Fw1	5′-CAACAGGACGGGCTGGTGAGG-3′
	RH2-2Rev3	5′-ACCCATTCCAATTGTTGCC-3′
RH1	RH1Fw1	5′-ATGAACGGCACAGAGGGACC-3′
	RH1Fw4	5′-GCAGTGCTCATGCGGAGTC-3′
	RH1Rev2	5′-CCTGTTGCTCCATTTATGCAGG-3′
	RH1Rev4	5′-GCTGGAGGACACAGAAGAGG-3′
LWS	Fw100	5′-GATCCCTTTGAAGGACCAAACT-3′
	Fw1a	5′-TCTTATCAGTCTTCACCAACGG-3′
	Gamma Fw1	5′-TGCTATGCAGCAGATAAATTG-3′
	RevEnd	5′-TTATGCAGGAGCCACAGAGG-3′
	Rev8	5′-GCCCACCTGTCGGTTCATGAAG-3′
	RevEx4	5′- CTTCCACTGAACACATCAGG-3′

Primers were used to amplify sequences from *A. anableps* cDNA and genomic DNA as well as guppy cDNA.

**Table 2:** Southern blot results.

Probe	Restriction Enzyme	Number of Bands	Band Size (Kb)
LWS	EcoRI	3	4.3, 4.1, 3.8
LWS	BamH1	3	5.0, 4.2, 4.0
SWS1	EcoRI	2	4.0, 2.0
SWS1	BamH1	1	4.1
RH2	EcoRI	1	2.5
RH2	BamH1	0	-
RH1	EcoRI	2	4.5, 4.0
RH1	BamH1	2	4.7, 3.8
SWS2	EcoRI	1	2.0
SWS2	BamH1	1	3.0

Summary of Southern blot analysis results obtained for *A. anableps* opsins probes with genomic DNA hybridized at 41°C. If Southern blot bands out-numbered unique cDNA sequences, we surveyed genomic DNA and sequenced at least five clones. Bands are pictured in Figure S1.

For the LWS opsin subfamily, two rounds of genomic PCR and sequencing were undertaken to supplement the original cDNA screen. The first round amplified the S180α gene that had been retrieved from cDNA and seven novel sequences. However, we suspected several to be mosaics produced during PCR (i.e., template switching) and/or during cloning (e.g., mismatch repair of cloned heteroduplex DNA) [25], [26]. In the second PCR survey of genomic DNA, LWS opsin primers were added at the beginning and then again just before the last PCR cycle in an attempt to eliminate these artefacts [26], [27]. Only genes uncovered in both rounds were considered to be authentic. These genes include LWS S180α, LWS S180β and LWS S180γ and an allele of LWS S180α. Subsequently, LWS S180γ was successfully amplified from cDNA.

### Phylogenetic analyses of A. anableps opsin genes

**Figure 1:**
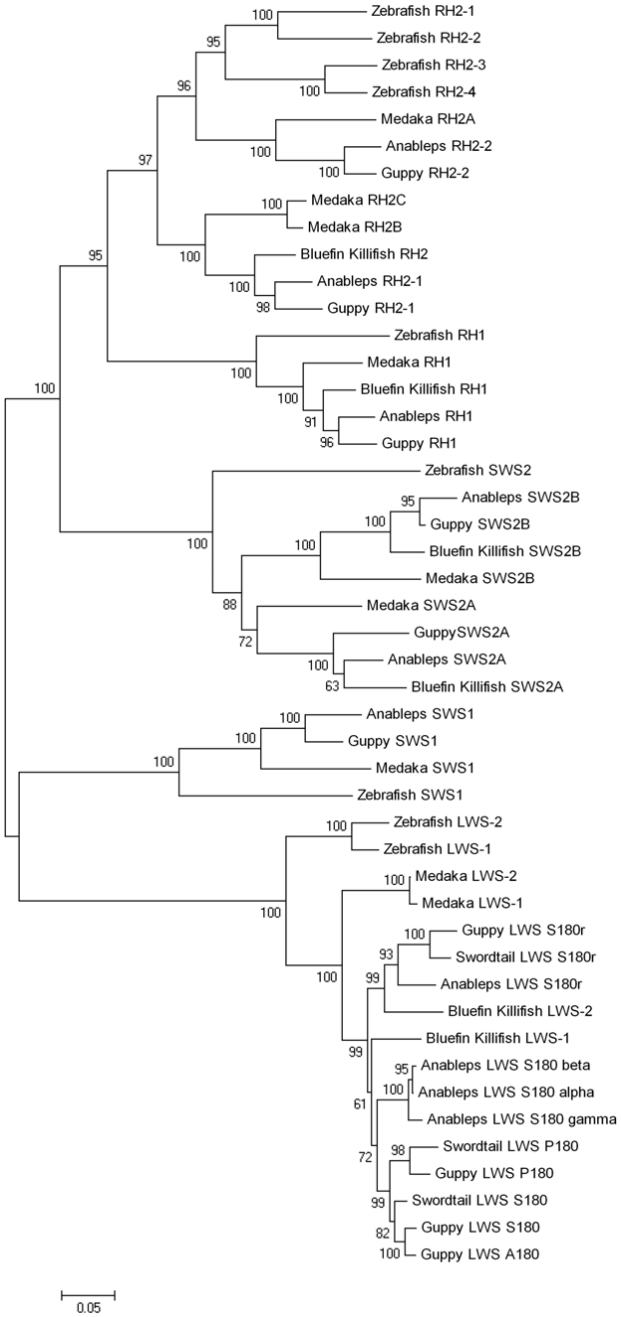
Phylogenetic analysis of *A. anableps* opsins. A neighbour-joining bootstrap consensus tree of visual opsins from *A. anableps* and its relatives. The percentage of trees in which the associated taxa clustered together in the bootstrap test (500 replicates) is shown at the nodes. Tamura-Nei algorithm was used and all codon positions were included. Missing nucleotides were treated with pairwise deletion in the analysis. Sequence accession numbers listed in Table 3.

All *A. anableps* opsin sequences were aligned with representatives of each subfamily from other fish species. Sequences in the alignment were 412 to 819 bp long (Table 3). We used Mega4 [28] to calculate Tamura-Nei genetic distances [29] and to reconstruct a neighbour joining tree (Figure 1). Sequences from each opsin subfamily formed well-supported monophyletic groups, with bootstrap support (500 replicates) ≥97%. Relationships among species within each opsin subfamily were consistent with well-established taxonomy [6], [30]. The root of the tree was positioned along the branch separating the LWS opsins from all others. While no non-opsin out-group sequences were employed in these analyses, the placement of the root between the LWS and all other subfamilies has been well established [31]. The *A. anableps* sequences occurred in each of the subfamilies confirming that locus-specific primers had amplified the genes they targeted. Phylogenetic analysis revealed an SWS2 gene duplication event that occurred in the common ancestor of bluefin killifish, *A. anableps* and guppy, although one of the duplicates had not been amplified from guppy and is reported here for the first time.

**Table 3:** Sequences used in phylogenetic analysis.

Common name	Scientific names	Gene name	Accession number
The “Four-eyed” Fish	*Anableps anableps*	LWS S180α	FJ11154
		LWS S180β	FJ11158
		LWS S180γ	FJ11157
		LWS S180r	FJ11155
		SWS1	FJ11153
		SWS2A	FJ11152
		SWS2B	FJ11151
		RH2-1	FJ11149
		RH2-2	FJ11150
		RH1	FJ11156
Guppy	*Poecilia reticulata*	LWS S180	EU329434
		LWS A180	EU329442
		LWS P180	EU329456
		LWS S180r	EU329457
		SWS1	DQ234861
		SWS2A	FJ11159
		SWS2B	DQ234860
		RH2-1	DQ234859
		RH2-2	DQ234858
		RH1	DQ912024
Swordtail	*Xiphophorus pygmaeus*	LWS S180	EU329481
		LWS P180	EU329478
		LWS S180r	EU329479
Bluefin Killifish	*Lucania goodei*	LWS-1	AY296740
		LWS-2	AY296741
		SWS1	AY296735
		SWS2A	AY296737
		SWS2B	AY296736
		RH2-1	AY296739
		RH1	AY296737
Medaka	*Oryzias latipes*	LWS-1	AB223051
		LWS-2	AB223052
		SWS1	AB223058
		SWS2A	AB223056
		SWS2B	AB223057
		RH2a	AB223053
		RH2b	AB223054
		RH2c	AB223055
		RH1	AB180742
Zebrafish	*Danio rerio*	LWS-1	NM131175
		LWS-2	NM001002443
		SWS1	BC060894
		SWS2	NM131192
		RH2-1	NM131253
		RH2-2	NM182891
		RH2-3	NM182892
		RH2-4	NM131254
		RH1	BC05288

Common name, scientific name, gene name and GenBank accession number for all sequences used in phylogenetic analysis.

### Variation at amino acid positions known to influence spectral sensitivity

We hypothesized that *A. anableps* opsins would contain unique amino acid substitutions to accompany its unusual eye morphology. However, with two exceptions (SWS2A and LWS S180γ), the key-site haplotypes in *A. anableps* visual opsins also occur in other fish with ‘normal’ eyes. The residues at all of the 12 key sites in the SWS2A opsin have been seen in other fish, but the entire haplotype found in *A. anableps*, appears to be unique. This haplotype is unlikely to produce a significant shift in maximal absorption according to mutagenesis analyses [32]. The *A. anableps* opsin gene, LWS S180γ, also has a unique five key-site haplotype. The fourth key site substitution (T285A) switching SHYTA to SHYAA is predicted to shift the λ_max_−16 nm [33].

## Discussion

A PCR-based survey of cDNA uncovered eight different opsin genes in *Anableps anableps*. Southern blotting, which utilized much longer probes than the PCR primers, indicated that additional genes might exist in the RH1, SWS1 and LWS opsin gene subfamilies. A subsequent PCR-based survey of genomic DNA uncovered two additional LWS opsin genes leading us to conclude that *A. anableps* possess ten visual opsins: one violet-sensitive SWS1 opsin gene, two genes from the blue-sensitive SWS2 subfamily, two genes encoding green-sensitive opsins from the RH2 subfamily, four LWS or red-sensitive opsin genes, and an RH1 gene.

### Phylogenetics, gene duplication and key sites

Phylogenetic analysis showed that the two RH2 opsin genes in *A. anableps* are orthologs of RH2-1 and RH2-2, duplicates produced in the ancestor of guppy, medaka (*Oryzias latipes*), pufferfish (*Takifugu rubripes*), and stickleback (*Gasterosteus aculeatus*). Although we have no data on opsin gene location in *A. anableps*, the RH2 gene pair appears to be the product of a tandem duplication event as RH2-1 and RH2-2 are linked in medaka and pufferfish [34], [35]. The single band produced during Southern analysis of RH2 genes might be explained by the lack of a cut site between the tandem duplicates. The *A. anableps* and guppy SWS2 opsin gene duplicates reported here are orthologs of tandem duplicates found in medaka, called SWS2A and SWS2B [34]. SWS1 appears to be a single-copy gene in nearly all fish, including *A. anableps*. One exception is ayu (*Plecoglossus altivelis*), which contains a species-specific SWS1 opsin gene duplication [36]. We sequenced a single RH1 opsin from *A. anableps* cDNA. Only Conger eel (*Conger myriaster*) and scabbardfish (*Lepidopus fitchi*) have RH1 duplicates [37], [38]. The Southern blot analysis showed two bands for RH1 and SWS1. This banding might have been caused by allelic variation in cut site loci or non-specific Southern probe hybridization.

**Figure 2:**
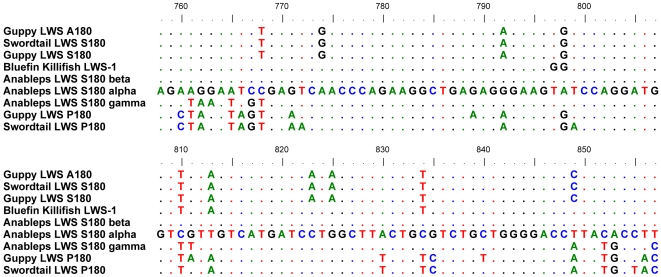
Sequence comparison between LWS genes. An alignment of 100 bp region of interest between between *A. anableps* LWS S180α and LWS S180γ. Over this area, *A. anableps* LWS S180γ is more similar to poeciliid P180, than to other *A. anableps* sequences.

LWS opsin gene duplication events have occurred independently in several fish lineages; the ancestors of zebrafish, medaka, guppy and blind cavefish (*Astyanax fasciatus*) each experienced independent LWS gene duplication events [11], [34], [39], [40]. In some cases, LWS opsin gene duplication has been followed by amino acid substitutions at sites known to influence spectral sensitivity (human, guppy and zebrafish), whereas in others (medaka, blind cavefish and bluefin killifish) duplicates have the same 5- key-site haplotype [11], [34], [39]–[42]. The single exon LWS opsin gene S180r, which, appears to have been produced by retrotransposition in the ancestor of the livebearers and bluefin killifish [11], has been retained in *A. anableps*. This gene is one of those that retained the SHYTA five key site haplotype after duplication. LWS P180 (with the key-site haplotype PHFAA) and LWS S180 (with key site haplotype SHYTA) are tandem duplicates that have been uncovered in *Poecilia* and *Xiphophorus* [11], two genera in the family Poeciliidae, sister family to Anablepidae [30]. However, the duplication event producing this gene pair appears to pre-date the poeciliid, anablepid divergence. The *A. anableps* LWS S180α gene is similar to, and has the same key-site haplotype as, guppy LWS S180 and the *A. anableps* LWS S180γ· gene contains a region at the 3′ end that is remarkably similar to the homologous portion of the poeciliid LWS P180 opsin gene (Figure 2). However, LWS S180γ is not the sister sequence to the poeciliid LWS P180 genes as predicted by the hypothesis that they are products of the same tandem duplication event. We believe this is a consequence of post-duplication gene conversion events within the genus *Anableps*. Thus it appears that a combination of duplication and conversion have produced a unique opsin (with a SHYAA haplotype) in a fish with unique eye morphology.

A recent duplicate present in *A. anableps* and not in close relatives is LWS S180β. It is identical to LWS S180α aside from a short region of sequence variation in the 5′ end. This variation changes the amino acid sequence; however, it does not result in a new key site haplotype as it occurs before amino acid position 180, the first of the five key sites in LWS opsins.

Thus, molecular adaptations for aerial vision, at the primary sequence level, may be confined to one of the most recently created opsin genes, LWS S180γ which has a unique key site haplotype (SHYAA instead of SHYTA), and coincides with the evolution of the unique eye morphology. Alternatively, these observed sequence level changes could be neutral or possibly even mildly deleterious. However, it is likely that spectral tuning through amino acid substitutions is just one part of the adaptations for aerial vision, along with eye morphology, photoreceptor distribution and opsin expression pattern changes [4], [43], [44].

### Implications for expression

Although little is currently known about opsin expression patterns in the *A. anableps* retina, we predict that the photic contrast between the aerial and aquatic environment will have provided a selective pressure for divergent patterns of expression. The turbid water that *A. anableps* lives in filters light, allowing the long wavelength light to transmit most readily [45]. It is possible that *A. anableps* copes with the differences in light composition by using different opsin expression patterns in its two retinal hemispheres. Previous MSP work has attempted to measure pigment differences between retinal hemispheres, but no differences in pigments present were detected [17]. However, as mentioned previously, MSP suggests *A. anableps* possessed only three visual pigments altogether. Although nine of the ten opsins in this study were recovered from cDNA and thus expressed to some level, there can be extreme variation in opsin expression levels, both between duplicates and during development, therefore it is possible that at any given time only a portion of the repertoire is functionally significant [46], [47]. In future studies, we will use *in situ* hybridization to examine the mechanism of visual adaptation in *A. anableps* using probes designed from the opsin repertoire characterized here. By cataloguing its opsin repertoire we have laid the groundwork for much exciting research in not only *A. anableps* itself, but in the nature of aerial and aquatic vision.

## Materials and Methods

### RNA isolation, cDNA synthesis and DNA isolation

Live *Anableps anableps* were obtained from a commercial supplier (The Afishionados, Winnipeg, Manitoba, Canada). One juvenile individual was euthanized in buffered MS222. Total RNA was isolated from one eye using Aurum™ Total RNA Fatty and Fibrous Tissue Pack, immediately after euthanasia and enucleation. RNA was stored at −80°C. cDNA was synthesized using BioRad® iScript Select cDNA Synthesis Kit from total RNA. DNA was isolated from muscle tissue using QIAquick® DNeasy Blood & Tissue Kit.

### Primer design and PCR

PCR primers were developed for eight genes in five visual opsin subfamilies, SWS1, SWS2, RH1, RH2, and LWS (Table 1). These primers were complementary to regions in each opsin gene or subfamily that were conserved in guppy (*Poecilia reticulata*), and bluefin killifish (*Lucania goodei*). Two forward and two reverse primers were employed for each gene.

Each primer pair was used to survey cDNA or genomic DNA in PCR reactions using Bio-Rad iProof high-fidelity DNA polymerase in an Eppendorf™ Mastercycler® EP Grad S thermocycler using the following conditions: Initial denaturation at 98°C for 30 seconds, 35 cycles with denaturation at 98°C for 5 seconds, annealing at 50–70°C (in 5°C gradations) for 12 seconds, extension at 72°C for 25 seconds and a final extension at 72°C for 5 minutes. During the second round of genomic DNA screening we added additional primers (1 µl at 10 mM) at the beginning of the last PCR cycle to prevent heteroduplex formation. Guppy cDNA was also surveyed using SWS2A opsin primers designed from conserved regions in *A. anableps* and bluefin killifish. The guppy PCR templates were obtained from a lab-reared fish descended from samples collected in Cumana, Venezuela (i.e., an Endler's guppy).

### Cloning

PCR products were run on 1.5% agarose gel. Amplicons of the predicted size were excised using QIAquick® Gel Extraction Kit. If only one band was observed the portion of the product not run on the gel was purified using QIAquick® PCR Purification Kit. Purified products were A-tailed using Invitrogen™ Taq polymerase and cloned using the Promega® pGEM™ - T Easy Vector System II kit. Clones containing inserts of the correct size were sequenced using labelled M13 forward and reverse primers and a Licor sequencer at the Centre for Biomedical Research at the University of Victoria.

### Southern Blotting

*A. anableps* genomic DNA was extracted from muscle tissue using phenol-chloroform extraction. DNA was digested in two separate reactions for 48 hours at 37°C using restriction enzymes, *EcoRI* and *BamHI*. Digestion was followed by overnight ethanol precipitation. Neither *EcoRI* nor *BamHI* cut within the region of the opsin genes that were complementary to the probes. 10 to 20 µg of digested DNA was electrophoresed in a 1.5% agarose gel and transferred onto a Bio-Rad® Zeta-Probe nylon membrane using the Bio-Rad® Model 785 Vacuum Blotter. Transferred DNA was immobilized by UV exposure for 5 minutes using a UVP HL-2000 HybriLinker prior to hybridization. DIG-labelled probes complementary to *A. anableps* opsins were synthesized using a Roche® PCR DIG Probe Synthesis Kit under the following amplification conditions: initial denaturation at 95°C for 2 minutes, 38 cycles with denaturation at 95°C for 30 seconds, annealing at 50–56°C for 30 seconds, extension at 72°C for 40 seconds and a final extension at 72°C for 7 minutes. These LWS, SWS1, SWS2, RH2, RH1 probes were amplified from cloned genomic DNA using the primer sets Fw100/Rev_Ex4, SWS1 Fw1/SWS1 Rev1, SWS2Fw3/SWS2Rev4, RH2 Fw1/RH2 Rev4 and RH1Fw4/RH1Rev4 (Table 1). Southern blot hybridization and detection was conducted according to the protocol provided in the Roche® DIG Application Manual for Filter Hybridization. Overnight hybridization at 41°C was performed in roller bottles using a UVP® HL-2000 HybridLinker. Hybridized membranes were subsequently washed at room temperature for 10 minutes (2×5 minutes) with 2× SSC followed by a 65°C wash for 30 minutes (2×15 minutes) with 0.5× SSC (both solutions contained 0.1% SDS). Roche® sheep Anti-Digoxigenin-AP, Fab fragments conjugated to alkaline phosphatase in conjunction with Roche® CSPD chemiluminescent substrate was used to detect the presence of bound digoxigenin probes. Generated blots were exposed to Roche® Lumi-film Chemiluminescent Detection Film for 3 to 24 hours prior to development.

### Phylogenetic analysis

A phylogenetic tree was reconstructed for the complete set of opsin sequences. It included sequences from guppy, swordtail, bluefin killifish, medaka and zebrafish (Table 3). Phylogenetic trees were constructed using Mega4 utilizing Tamura-Nei algorithm, Neighbour-joining, and support for nodes were estimated using 500 bootstrap reanalyses [28], [29], [48], [49]. Sequences were 412 to 819 bp long.

## Supporting Information

Figure S1 Southern blot images. A composite image of all Southern blot results for A. anableps opsins probes with A. anableps genomic DNA hybridized at 41°C. Bands are indicated with arrows and quantified in Table 2.
(5.21 MB TIF)
