# Supplementary figures and images for: A Fish Eye Out of Water: Ten Visual Opsins in the Four-Eyed Fish, Anableps anableps
Source: PLoS One. 2009 Jun 24;4(6):e5970. doi: 10.1371/journal.pone.0005970 (PMC2696081; doi:10.1371/journal.pone.0005970)

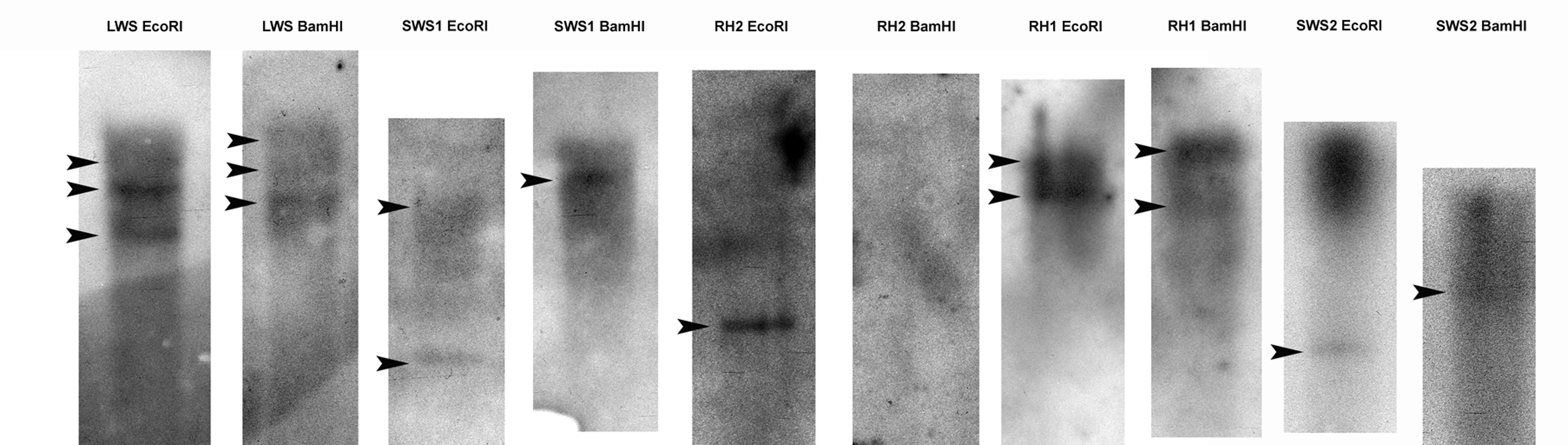

Supplement: Figure S1 — Southern blot images. A composite image of all Southern blot results for A. anableps opsins probes with A. anableps genomic DNA hybridized at 41°C. Bands are indicated with arrows and quantified in Table 2. (5.21 MB TIF) [file pone.0005970.s001.tif]
